# The effects of secreted aspartyl proteinase inhibitor ritonavir on azoles‐resistant strains of *Candida albicans* as well as regulatory role of *SAP2* and *ERG11*

**DOI:** 10.1002/iid3.415

**Published:** 2021-05-05

**Authors:** Wenli Feng, Jing Yang, Yan Ma, Zhiqin Xi, Xiaoqin Zhao, Xiaoxia Zhao, Min Zhao

**Affiliations:** ^1^ The Department of Dermatovenereology The Second Hospital of Shanxi Medical University Taiyuan Shanxi China

**Keywords:** *Candida albicans*, ERG11, SAP2, secreted aspartyl proteinases

## Abstract

**Background:**

*Candida albicans*, the main human fungal pathogen, can cause fungal infection and seriously affect people's health and life. This study aimed to investigate the effects of ritonavir (RIT) on *C. albicans* and the correlation between *SAP2* as well as *ERG11* and drug resistance.

**Results:**

Secreted aspartyl proteinases (Saps) activities and pathogenicity of *C. albicans* with different drug resistance were measured. M27‐A4 broth microdilution method was used to analyze the drug sensitivity of RIT combined with fluconazole (FCA) on *C. albicans*. After that, *SAP2* and *ERG11* mutations were examined by polymerase chain reaction (PCR) and sequencing, and quantitative real‐time PCR was utilized to determine the expression of the two genes. By analyzing *p*z values, the Saps activity of cross‐resistant strains was the highest, followed by voriconazole (VRC)‐resistant strains, FCA‐resistant strains, itraconazole (ITR)‐resistant strains, and sensitive strains. The pathogenicity of *C. albicans* in descending order was as follows: cross‐resistant strains, VRC‐resistant strains, ITR‐resistant strains, FCA‐resistant strains, and sensitive strains. With the increase of RIT concentrations, the Saps activity was gradually inhibited. Drug sensitivity results showed that there was no synergistic effect between RIT and FCA. Additionally, no gene mutation sites were found in *SAP2* sequencing, and 17 synonymous mutations and 6 missense mutations occurred in *ERG11* sequencing. Finally, the expression of *SAP2* and *ERG11* was significantly higher in the resistant strains compared with the sensitive strains, and there was a positive liner correlation between *SAP2* and *ERG11* messenger RNA expression (*r* = .6655, *p* < .001).

**Conclusion:**

These findings may help to improve our understanding of azole‐resistant mechanisms of *C. albicans* and provide a novel direction for clinical therapeutics of *C. albicans* infection.

## Graphical abstract

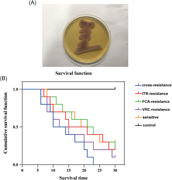

**Abbreviations**
C. albicans: Candida albicans
FCA: fluconazole
ITR: itraconazole
RIT: ritonavir
Saps: secreted aspartyl proteinases
VRC: voriconazole

## 1 BACKGROUND

*Candida albicans* is a common opportunistic fungus that usually colonized in the gastrointestinal tract, reproductive tract, mouth and skin, and can cause a variety of diseases, from superficial mucosal diseases to life‐threatening systemic diseases.^1^ Approximately 75% of women are likely to experience at least one vulvovaginal candidiasis event in their lifetime, and about 5% female population have recurrent infections.^2^ Additionally, deep infection may remain existed or contribute to secondary candidiasis, which can seriously endanger people's health.^3^ At present, the treatment of candidiasis mainly depends on antifungal drugs, such as polyenes, azoles and echinocandins.^4^ However, with the widespread use of antibiotics and the changes in the living environment of *C. albicans*,^5^ the morbidity and drug resistance of *C. albicans* are increasing year by year, which is a major problem in the clinical treatment of *C. albicans* infection. Therefore, it is vital to explore the pathogenic and drug‐resistant mechanisms of *C. albicans*, and to search for novel therapeutic strategies.

*C. albicans* has many pathogenic virulence properties, including the formation of biofilms, cell phenotypes transformation, adhesins on the cell surface and the expression of invasive proteases.^6^ Secreted aspartyl proteinases (Saps), an invasive protease, are the primary virulence factor of *C. albicans*, and play important roles in the infection and pathogenesis of *C. albicans*.^7^ Previous study had shown that Saps were closely related to the occurrence and development of vaginitis caused by *C. albicans*.^8^ Therefore, it is possible to find drugs targeting Saps for the treatment of *C. albicans* infection. A study of Dos Santos^9^ showed that a protease inhibitor ritonavir (RIT) significantly reduced the infectious rate of *C. albicans* in human immunodefeciency virus (HIV) patients, and indicated that RIT and Saps belonged to the aspartic protease family, which may be used as a new drug to inhibit Saps. Other studies have reported that 0.1 mg/ml RIT could significantly suppress adhesion of *C. albicans* to epithelial cells and acrylics,^10^ as well as 8 mg/L of RIT inhibited 44% of growth in *C. albicans*.^11^ When using RIT alone, due to the high concentration of RIT and the increase metabolic burden of human liver, it is necessary to adopt combination therapy against *C. albicans*.

*SAP2*, considered as a key virulence factor, can provide nutrients for its own growth by degrading macromolecular proteins on the mucosal surface, and simultaneously increase the ability of *C. albicans* to adhere and invade the host. Our previous research had found that the messenger RNA (mRNA) expression of *SAP2* in the itraconazole (ITR)‐resistant strains was higher than that in the ITR‐sensitive strains, which indicated that *SAP2* may be associated with the drug resistance of *C. albicans*.^12^ Furthermore, lanosterol 14 ‐demethylase (14‐DM/ERG11p), an important component in ergosterol synthesis, is the target enzyme of azole drugs, and is encoded by *Ergosterol 11* (*EGR11*).^13^ Azole drugs, such as fluconazole (FCA) achieved antifungal effects by binding with *ERG11* and inactivating the target enzymes. However, if *ERG11* mutates, the molecular configuration of the target enzyme ERG11p will change, thus reducing the affinity between azole drugs and ERG11p and leading to drug resistant. Suchodolski et al.^14^ found that the increased expression of *ERG11* in *C. albicans* contributed to the increased production of ergosterol, and promoted drug resistance of strains. However, the expression of *SAP2* and *ERG11* in *C. albicans* with different drug resistance and the relationship between *SAP2* as well as *ERG11* and drug resistance remain unclear.

In this study, clinical *C. albicans* strains with different drug resistance were used, and the Saps activities in different drug resistant strains were measured in vitro and in vivo. After that, the effects of RIT on Saps activities in *C. albicans* were determined, and the synergistic effects of FCA combined with RIT on *C. albicans* were investigated. Further, *SAP2* and *ERG11* in different drug resistant strains were sequenced, and the relationship between *SAP2* as well as *ERG11* and drug resistance was explored. These findings will provide a theoretical basis for controlling the occurrence of drug resistance, and provide a new direction for clinical therapeutics of diseases caused by *C. albicans*.

## 2 METHODS

### 2.1 Experimental strains

A total of 50 clinical *C. albicans* strains isolated and identified by the Dermatovenereal Fungus Laboratory of The Second Hospital of Shanxi Medical University were used. Among them, there were 10 FCA‐resistant strains, 10 ITR‐resistant strains, 10 voriconazole (VRC)‐resistant strains, 10 FCA, ITR, or VRC‐sensitive strains, and 10 FCA, ITR and VRC cross‐resistant strains. Additionally, standard strain *C. albicans* ATCC1106, and quality control strains *Candida krusei* ATCC6258 and *Candida parapsilosis* ATCC22019 were purchased from the Fungus and Mycosi Research Center, Peking University Medical Science.

### 2.2 Detection of Saps activity in vitro

Base on the methods of Li et al.^15^ and Barros et al.,^16^ the Saps activity was measured in vitro using bovine serum albumin (BSA) agar medium. Briefly, 50 clinical strains were inoculated in Sabouraud dextrose agar (SDA) Medium (Beijing Luqiao Technology Co. Ltd), and cultured at 25°C for 48 h. After that, a fresh single colony was selected, and the fungal suspension was adjusted to 1 × 10^7^ CFU/ml with sterile saline. Afterwards, fungal suspension (5 μl) was inoculated to the center of the BSA agar medium (Beijing Luqiao Technology Co. Ltd), or the BSA agar medium containing 32, 8, 2, and 0 μg/ml RIT. After cultured at 37°C for 72 h, the size of colony ring and surrounding transparent ring were examined. The Saps activity was evaluated by the ratio of colony diameter to diameter of the dense white zone of precipitation around phospholipase (PL) positive colonies (*p*z value).^17^ The formula of *p*z value was shown as follows: *p*z value = (colony diameter)/(colony diameter + the diameter of the transparent ring). The experiment was repeated three times for each strain.

### 2.3 In vivo pathogenicity of *C. albicans* with different drug resistance

A total of 60 SPF female mice with 6 weeks old were obtained from the Animal Center of Shanxi Medical University. All mice were free to drink and eat during the experiment. The mice were fed under controlled temperature (24 ± 2°C) and humidity (50 ± 5%) conditions, with a 12 h light/dark cycle. After 3 days of adaptive feeding, the mice were randomly divided into six groups (*n* = 10): cross‐resistant group, FCA‐resistant group, ITR‐resistant group, VRC‐resistant group, sensitive group, and control group. The clinical strains with different drug resistance were inoculated in SDA medium, and cultured at 25°C for 48 h. Sterile saline was used to adjust fungal suspension to 1 × 10^7^ CFU/ml. The mice in the cross‐resistant group, FCA‐resistant group, ITR‐resistant group, VRC‐resistant group, and sensitive group were injected with 0.2 ml cross‐resistant fungal suspension, FCA‐resistant fungal suspension, ITR‐resistant fungal suspension, VRC‐resistant fungal suspension, and sensitive fungal suspension, respectively, via tail vein.^12^ The mice in the control group were injected with 0.2 ml saline into caudal vein. The appetite and mental state of the mice were observed three times a day. After observed for 30 days, the mortality and survival rates were counted.

After the experiment, all the mice were killed by cervical dislocation. The protocol was approved by the Ethics Committee of the Second Hospital of Shanxi Medical University (approval number: [2019]YX[24D]).

### 2.4 Drug sensitivity assay under different conditions

The purified *C. albicans* was resuspended in the RPMI1640 medium (Beijing Luqiao Technology Co. Ltd), and the fungal suspension was adjusted to 5 × 10^3^ CFU/ml by the RPMI1640 medium. The *C. albicans* suspension in a free state was prepared. The preparation of *C. albicans* suspension under biofilm condition was shown as follows. The fungal suspension was adjusted to 5 × 10^6^ CFU/ml by the RPMI1640 medium. The suspension (100 μl) was added to each well of a 96‐well plate (except the negative control), and cultured at 37°C for 1 h. After washing with phosphate‐buffered saline (PBS) twice, 100 μl RPMI1640 medium was added, and then cultured at 37°C for 24 h. After washing with PBS three times, the medium was discarded, and the fungal suspension was cultured for another 24 h to form mature biofilm.

The sensitivity of RIT combined with FCA on *C. albicans* was determined using the Clinical and Laboratory Standards Institute (CLSI) standard M27‐A4 broth microdilution method as previously described.^18^ The concentrations of FCA were prepared in double distilled water from 0.125 to 128 μg/ml by a two‐fold dilution method; while the concentrations of RIT were prepared in dimethyl sulfoxide from 2 to 256 μg/ml. Under free conditions, each concentration of FCA (100 μl) was added to a 96‐well plate, and then each concentration of RIT (100 μl) was added. Afterwards, the fungal suspension was added to each well of the 96‐well plate. The control groups were added 100 μl PRMI1640 medium. Under biofilm conditions, the biofilm of *C. albicans* was firstly established in a 96‐well plate, and different concentrations of FCA or RIT (100 μl) was added. RPMI1640 medium was used as the control. After cultured at 37°C for 48 h, the minimal inhibitory concentration (MIC) values were read based on the CLSI.^19^ When MIC ≤ 2, the strains were identified as sensitive to FCA (s); when MIC = 4, the strains were dose‐dependent sensitive to FCA; when MIC ≥ 8, the strains were resistant to FCA (R).

The interaction between RIT and FCA on *C. ablicans* was analyzed based on the values of FICI.^20^, ^21^ The formula of FICI was shown as follows: FICI = (MIC_FCA_ in combination/MIC_FCA_ alone) + (MIC_RIT_ in combination/MIC_RIT_ alone). If FICI ≤ 0.5, FCA and RIT have synergistic effects; if 0.5 < FICI ≤ 1, FCA and RIT have additive effects; if 1 < FICI < 4, there is no interaction between FCA and RIT; if FICI ≥ 4, RIT and FCA have antagonistic effects.

### 2.5 DNA extraction, and SAP2 and ERG11 sequencing

DNA was extracted from *C. albicans* using OMEGA D3370 yeast DNA extraction kit (Guangzhou Feyou Biotechnology Co., Ltd) following the manufacturer's instructions. The primer sequences of *ERG11* and *SAP2* were shown in Table 1, and synthesized by Sangon Biotech (Shanghai) Co., Ltd. The volume of polymerase chain reaction (PCR) system was 25 μl, including 2X TaqPCR Mix 12.5 μl, forward primer 1 μl, reverse primer 1 μl, DNA 2.5 μl, and double distilled water 8 μl. The PCR reaction was initiated at 94°C for 10 min, followed by 94°C for 45 s, 52°C/48.5°C (*SAP2*/*ERG11*) for 45 s, and 72°C for 2 min, for a total of 30 cycles, and finally ended at 72°C for 10 min.

**Table 1:** The primer sequences of *ERG11* and *SAP2*

**Process**	**Primer**	**Sequence (5′‐3′)**	**Gene code**	**Length (bp)**
PCR	SAP2	F: ATGTTTTTAAAGAATATTTTCATTGCTCTTGC	NC032096	1197
		R: TTAGGTCAAGGCAGAAATACTGGA		
	ERG11	F: CAAGAAGATCATAACTCAAT	X13296	1641
		R: CAGAACACTGAATCGAAAGA		
RT‐qPCR	SAP2	F: TGGTATTCTTATGGGTGGTC	/	357
		R: TTAGCAGCAGCAGTATCC		
	ERG11	F: CAAGTGGTTCATCAGCTTCAC	/	272
		R: TTATTTGTCCCGTGGCAG		

Abbreviations: PCR, polymerase chain reaction; RT‐qPCR, quantitative real‐time PCR.

The PCR products were purified and two directional sequenced by Sangon Biotech (Shanghai) Co., Ltd. The gene sequences of the PCR products were analyzed by Blast software, and then were compared with the known sequences in GenBank database (NC032096/X13296). Additionally, the sequences are translated into amino acid sequences to identify gene mutation sites by chromas and secentral softwares.

### 2.6 RNA extraction and real‐time quantification PCR

According to the manufacturer's protocols, column yeast total RNA extraction and purification kit (Sangon Biotech (Shanghai) Co., Ltd) utilized to isolate total RNA from *C. albicans*. Afterwards, the concentration and quality of total RNA were measured using a microplate reader. Subsequently, RNA was reverse‐transcribed to cDNA using a cDNA synthesis kit (Roche Diagnostics Products Co., Ltd.) following the manufacturer's recommendations. The primer sequences of ERG11 and SAP2 were shown in Table 1. The quantitative real‐time PCR (RT‐qPCR) reaction was begun at 95°C for 10 min, followed by a total of 45 cycles at 95°C for 10 s, 55°C for 15 s and 72°C for 20 s. ACT1 was served as a housekeeping gene, and the relative expression levels of *ERG11* and *SAP2* was calculated using the $2^{‐∆∆C_{t}}$method.^22^

### 2.7 Statistical analysis

Each experiment repeated three times (*n* = 3). SPSS 22.0 software (SPSS, Inc.) was utilized for statistical analyses. Data were expressed as mean ± *SD*) The Log‐rank test in Kaplan–Meier was used to compare the survival time among the groups. One‐way analysis of variance was applied for the comparison of multiple groups. *p* < .05 was considered as statistical significance. The correlation between *SAP2* expression and *ERG11* expression was analyzed using Pearson linear correlation coefficient.

## 3 RESULTS

### 3.1 Saps activity analysis of *C. albicans* in vitro

**Figure 1:**
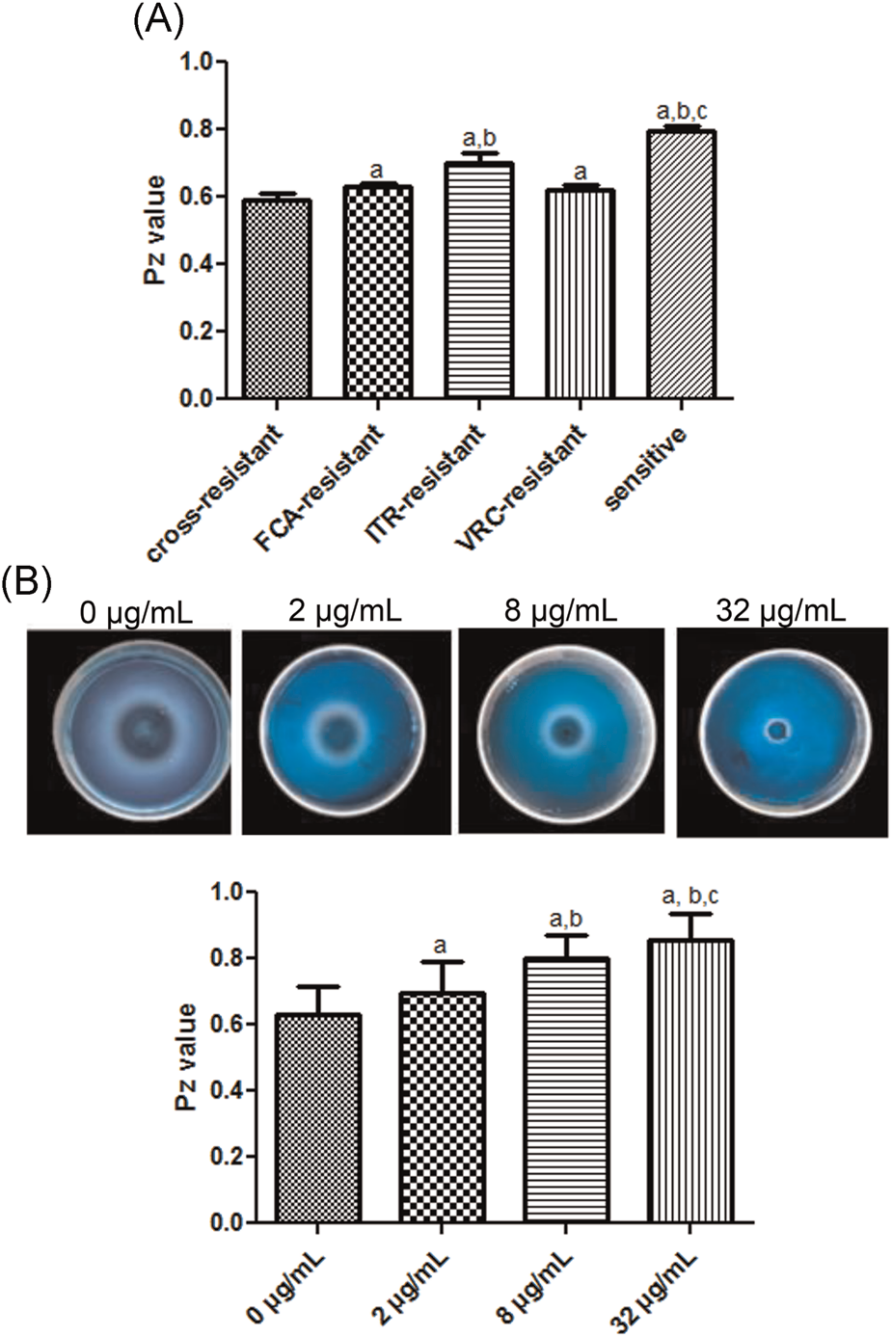
(A) *p*z values of *Candida albicans* with different drug resistance. a: *p* < .05, compared with the cross‐resistant strains. b: *p* < .05, compared with the FCA‐resistant strains. c: *p* < .05, compared with the ITR‐resistant strains. (B) Secreted aspartyl proteinases (Saps) activity of *C. albicans* treated with different concentrations of ritonavir (RIT). Upper: The formation of colony ring and transparent ring in *C. albicans* with different concentrations of RIT. Lower: *p*z value of *C. albicans* with different concentrations of RIT. a: *p* < .05, compared with the strains treated with 0 μg/ml RIT. b: *p* < .05, compared with the strains treated with 2 μg/ml RIT. c: *p* < .05, compared with the strains treated with 8 μg/ml RIT. FCA, fluconazole; ITR, itraconazole; VRC, voriconazole

The Saps activity of all experimental strains with different drug resistance was determined. The *p*z values of cross‐resistant strains, FCA‐resistant strains, ITR‐resistant strains, VRC‐resistant strains and sensitive strains were 0.59 ± 0.023, 0.631 ± 0.01, 0.701 ± 0.028, 0.623 ± 0.011, and 0.795 ± 0.016, respectively. By the analysis of variance, there were significant differences of *p*z values among the cross‐resistant strains, FCA‐resistant strains, ITR‐resistant strains, VRC‐resistant strains and sensitive strains (*p* < .05, Figure 1A). The results indicated that the Saps activity of cross‐resistant strains was the highest, followed by the VRC‐resistant strains, FCA‐resistant strains, ITR‐resistant strains, and sensitive strains.

Subsequently, different concentrations of RIT were used to treat *C. albicans*, and the effects of RIT on Saps activity were evaluated. It was obvious that all *C. albicans* formed transparent rings, that is, all these strains produced Saps (Figure 1B). Compared with the control group (0 μg/ml), the *p*z values in the strains treated with RIT were gradually increased with the increase of RIT concentrations (*p* < .05, Figure 1B). This suggested that with the increase of RIT concentrations, the Saps activity of *C. albicans* could be suppressed.

### 3.2 **P**athogenicity analysis of *C. albicans* with different drug resistance in mice

**Figure 2:**
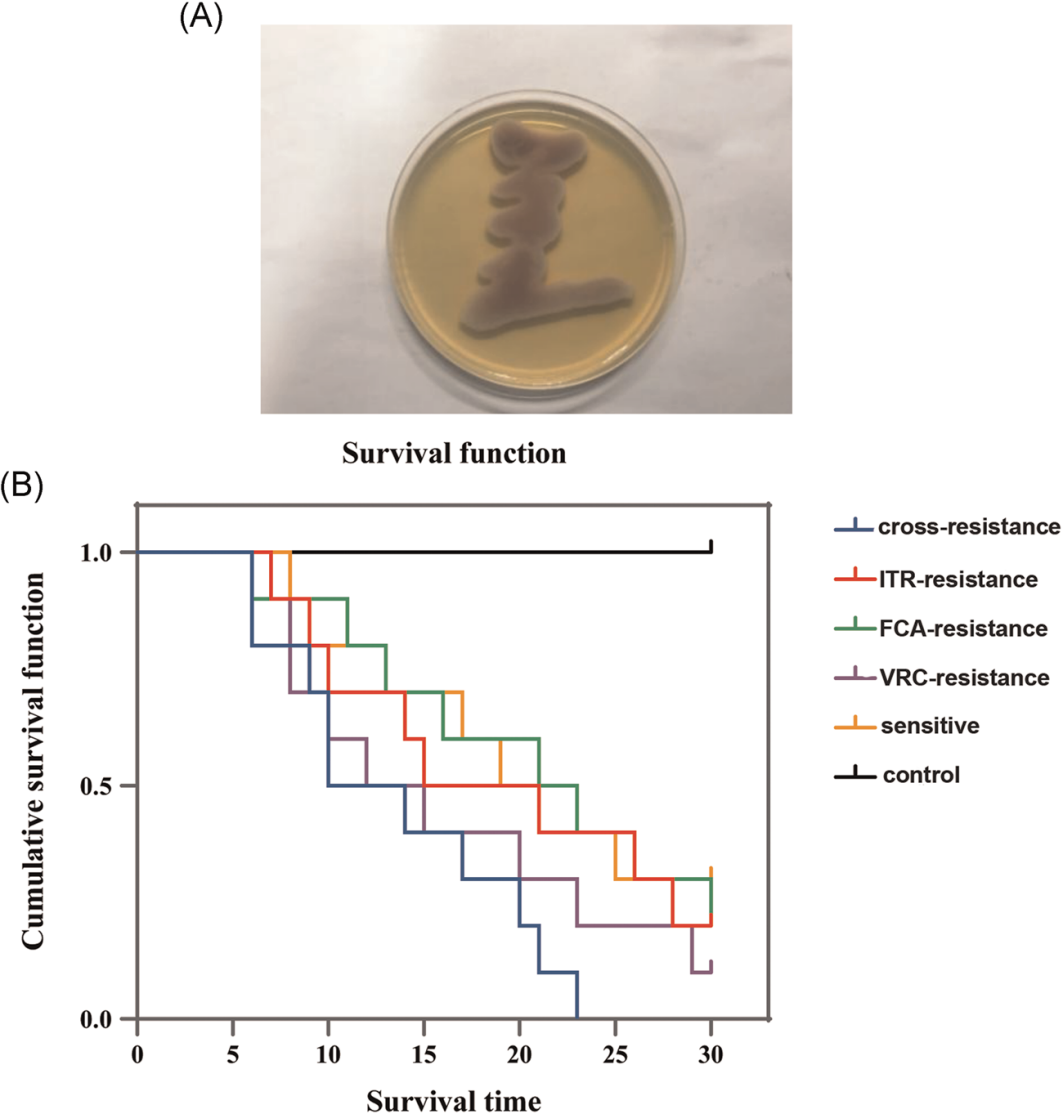
(A) The blood culture result of mice treated with *C. albicans* on Sabouraud dextrose agar medium at 37°C for 48 h. (B) Survival curves of mice treated with *C. albicans* with different drug resistance. FCA, fluconazole; ITR, itraconazole; VRC, voriconazole

The mice blood was cultured in SDA medium after the mice treated with *C. albicnas*, including cross‐resistant, FCA‐resistant, ITR‐resistant, VRC‐resistant and sensitive strains. After cultured at 37°C for 48 h, smooth milky white cheese‐like colonies were found (Figure 2A), which indicated that the fungemia mice model was successfully established.^23^ There was no death in the control group during a 30‐day observation period. The median survival time of the cross‐resistant group, FCA‐resistant group, ITR‐resistant group, VRC‐resistant group, and sensitive group was 12, 22, 18, 13.5, and 20 days, respectively (Table 2). The survival rates at 10, 20, and 30 days in different groups were shown in Table 2. In addition, the survival curves showed that the survival rate in escalating order was as follows: cross‐resistant group, VRC‐resistant group, ITR‐resistant group, FCA resistant group and sensitive group (Figure 2B). The results indicated that the pathogenicity of cross‐resistant strains was the highest, followed by VRC‐resistant strains, ITR‐resistant strains, FCA‐resistant strains, and sensitive strains.

**Table 2:** Median survival time and survival rate of mice in each group

**Groups**	**Median survival time (day)**	**Survival rate (%)**			**Log‐rank test**	
		**10 Day**	**20 Day**	**30 Day**	***χ* ^**2**^**	***P***
Cross‐resistant	12	50	20	0	25.62	0.00
FCA‐resistant	22	90	60	20		
ITR‐resistant	18	70	50	20		
VRC‐resistant	13.5	60	30	10		
Sensitive	20	80	50	30		
Control	30	100	100	100		

Abbreviations: FCA, fluconazole; ITR, itraconazole; VRC, voriconazole.

### 3.3 Drug sensitivity under free and biofilm conditions

M27‐A4 broth microdilution method was carried out to determine the drug sensitivity of FCA combined with RIT on *C. albicans* under free and biofilm conditions. The MIC_50_ values of FCA combined with RIT under free and biofilm conditions were presented in Table 3. By analyzing FICI, it was found that under free conditions, additive effects of FCA and RIT were observed in 8 strains with 0.5 < FICI ≤ 1, and no interaction between FCA and RIT was shown in 42 strains with 1 < FICI ≤ 4 (Table 4). However, under biofilm conditions, additive effects of FCA and RIT were shown in 12 strains, and no interaction was in 38 strains (Table 4). These results showed that RIT and FCA had no synergistic effects on *C. albicans* with different drug resistance.

**Table 3:** The minimal inhibitory concentration (MIC_50_) of fluconazole (FCA) combined with ritonavir (RIT) under free and biofilm conditions (unit: g/ml)

**Number**	**Free conditions (MIC_50_)**				**Biofilm conditions (MIC_50_)**			
	**FCA**	**FCA/RIT**	**RIT**	**FICI**	**FCA**	**FCA/RIT**	**RIT**	**FICI**
CA‐1	0.5	0.5	>128	2	2	2	>256	2
CA‐4	2	2	>128	2	8	4	>256	1
CA‐6	64	16	>128	0.75	128	128	>256	2
CA‐7	32	32	>128	2	128	128	>256	2
CA‐9	64	64	>128	2	128	64	>256	1
CA‐10	4	4	>128	2	16	8	>256	1.5
CA‐11	2	2	>128	2	8	8	>256	2
CA‐12	2	0.5	>128	0.75	8	8	>256	2
CA‐14	2	2	>128	2	16	16	>256	2
CA‐15	2	2	>128	2	8	2	>256	0.75
CA‐18	64	64	>128	2	128	128	>256	2
CA‐20	0.12	0.12	>128	2	0.5	0.5	>256	2
CA‐21	2	2	>128	2	8	8	>256	2
CA‐22	1	1	>128	2	4	4	>256	2
CA‐23	4	1	>128	1.25	64	64	>256	2
CA‐24	16	16	>128	2	64	64	>256	2
CA‐25	2	2	>128	2	4	4	>256	2
CA‐26	64	64	>128	2	128	128	>256	2
CA‐27	0.5	0.25	>128	1.5	4	1	>256	0.75
CA‐28	1	1	>128	2	4	4	>256	2
CA‐29	4	4	>128	2	64	64	>256	2
CA‐30	0.25	0.25	>128	2	0.5	0.5	>256	2
CA‐31	2	0.5	>128	0.75	8	2	>256	0.75
CA‐32	32	16	>128	1.5	128	128	>256	2
CA‐33	4	2	>128	1	64	64	>256	2
CA‐35	0.5	0.5	>128	2	2	1	>256	1
CA‐37	8	8	>128	2	64	32	>256	1
CA‐38	2	1	>128	1.5	8	8	>256	2
CA‐40	16	16	>128	2	64	64	>256	2
CA‐41	8	8	>128	2	64	64	>256	2
CA‐42	4	2	>128	1	64	16	>256	0.75
CA‐45	8	8	>128	2	32	32	>256	2
CA‐46	64	32	>128	1	128	128	>256	2
CA‐48	16	16	>128	2	64	64	>256	2
CA‐49	2	2	>128	2	64	64	>256	2
CA‐52	32	32	>128	2	128	128	>256	2
CA‐54	64	64	>128	2	128	128	>256	2
CA‐56	64	32	>128	1	128	128	>256	2
CA‐58	0.5	0.25	>128	1.5	64	16	>256	0.75
CA‐59	64	32	>128	1	128	128	>256	2
CA‐60	8	8	>128	2	16	4	>256	0.75
CA‐61	2	1	>128	1	16	16	>256	2
CA‐63	8	8	>128	2	64	16	>256	0.75
CA‐66	0.5	0.25	>128	1.5	1	64	>256	1
CA‐72	64	64	>128	2	128	128	>256	2
CA‐77	1	1	>128	2	8	8	>256	2
CA‐89	0.25	0.25	>128	2	1	1	>256	2
CA‐90	4	2	>128	1.5	32	16	>256	1.2
CA‐94	32	32	>128	2	128	128	>256	2
CA‐99	0.25	0.25	>128	2	0.5	0.5	>256	2

**Table 4:** The sensitization rate of FCA combined with RIT under free and biofilm conditions

	**FCA combined with RIT**	
	**Free condition (rate)**	**Biofilm condition (rate)**
Synergistic (FICI ≤ 0.5)	0	0
Additive (0.5 < FICI ≤ 1)	8 (16%)	12 (24%)
No interaction (1 < FICI ≤ 4)	42 (84%)	38 (76%)
Antagonistic (FICI > 4)	0	0

Abbreviations: FCA, fluconazole; FICI, fractional inhibitory concentration index; RIT, ritonavir.

### 3.4 SAP2 and ERG11 sequencing analyses

*SAP2* gene sequencing results showed that among 50 strains of *C. albicans*, 45 strains were successfully sequenced, and no gene mutation sites were found in all sequencing strains.

For *ERG11* gene sequencing, 43 strains of *C. albicans* were successfully sequenced, and 35 strains had base mutations, including 17 kinds of synonymous mutations (C363T, T462C, A504G, C558T, T696C, C805T, T1143C, A1167G, A1173G, A1230G, C1257T, T1350C, T1431C, C1443T, T1449C, A1587G, T1617C) and 6 kinds of missense mutations (T495A, C515T, A530C, T541C, G1609A, G1693C). As shown in Table 5, these mutations corresponded to the mutations of six different amino acids (D116E, T123I, K128T, Y132H, V488I, A516P). There were no mutations for standard strain ATCC11006.

**Table 5:** Base mutation sites and amino acid substitution in *ERG11* gene of *C. albicans*

**Strains**	**Resistance to FCA/ITR/VRC**	**Base mutation sits**	**Amino acid substitution**
CA‐1	VRC	T1431C	No
CA‐6	FCA	C363T/T462C/C558T/T696C/C805T/A1587G	No
CA‐7	FCA	T462C/T495A/C558T/T1143C/A1230G/C1257T/T1431C/A1587G/T1617C	D116E
CA‐9	FCA/ITR/VRC	T462C/T495A/A530C/A1587G/T1617C	D116E/K128T
CA‐10	VRC	T1431C	No
CA‐11	ITR	T462C/T495A/A1167G/A1587G	D116E
CA‐14	S	T462C/T495A/A1587G	D116E
CA‐15	VRC	C363T/T462C/C558T/T696C/C805T/T1143C/A1173G/C1257T/T1350C/C1443T/T1449C/G1609A	V488I
CA‐18	FCA/ITR/VRC	T462C/T495A/C515T/C1257T/A1587G	D116E/T123I
CA‐20	S	C363T/C558T/C805T/A1167G/A1587G	No
CA‐23	VRC	A1167G/C1443T/A1587G	No
CA‐26	FCA/ITR/VRC	T462C/T495A/C558T/C1257T/T1350C/T1431C/A1587G/T1617C/G1693C	D116E/A516P
CA‐29	ITR	T462C/T495A/A530C/C558T/C1257T	D116E/K128T
CA‐30	VRC	T462C/T495A/A1230G/C1257T/T1350C/T1431C/A1587G/T1617C	D116E
CA‐31	ITR	C363T/T462C/C558T/C805T/A1167G/A1587G	No
CA‐32	FCA	T462C/T495A/A530C/A1587G	D116E/K128T
CA‐33	ITR	T462C/T495A/A1587G	D116E
CA‐35	ITR	C363T/T462C/C558T/T696C/C805T/T1143C/C1257T/T1350C/C1443T/T1449C/G1609A	V488I
CA‐37	FCA/ITR/VRC	T462C/T495A/C558T/C805T/T1143C/A1173G/C1257T/T1350C/C1443T/T1449C	D116E
CA‐38	VRC	T462C/T495A/A1587G	D116E
CA‐40	FCA/ITR/VRC	T462C/T495A/A530C/A1587G	D116E/K128T
CA‐41	FCA	T462C/T495A/A1167G/A1587G	D116E
CA‐42	ITR	A1167G/A1587G	No
CA‐45	FCA/ITR/VRC	C363T/T462C/A504G/C558T/T696C/C805T/A1587G	No
CA‐48	FCA	A1167G/C1257T/A1587G	No
CA‐49	ITR	A1587G	No
CA‐52	FCA/ITR/VRC	C363T/T462C/C558T/C805T/T1143C/A1173G/T1350C/C1443T/T1449C	No
CA‐54	FCA/ITR/VRC	C363T/T462C/C558T/T696C/C805T/A1173G/T1350C/T1449C/G1609A	V488I
CA‐56	FCA/ITR/VRC	T462C/T495A/A504G/A530C/T541C/C558T	D116E/K128T/Y132H
CA‐59	VRC	T462C/T495A/A1587G	D116E
CA‐60	FCA	C363T/T462C/C558T/C805T/T1143C	No
CA‐61	ITR	A1230G/T1431C	No
CA‐63	FCA	A1587G	No
CA‐66	ITR	T1431C	No
CA‐94	FCA	C363T	No

Abbreviations: A, alanine; D, aspartic acid; E, glutamic acid; FCA, fluconazole; FICI, fractional inhibitory concentration index; H, histidine; I, isoleucine; K, lysine; P, proline; RIT, ritonavir; S, sensitive to FCA, ITR and VRC; T, threonine; V, valine; Y, tyrosine.

In the sensitive strains, two strains showed synonymous mutations and one strains had missense mutations (T495A→D116E). In the drug resistant strains (cross‐resistant, FCA‐resistant, ITR‐resistant and VRC‐resistant strains), there were 17 synonymous mutations and 6 missense mutations. Among them, in the single drug resistant strains (FCA‐resistant, ITR‐resistant and VRC‐resistant strains), 11 of 24 strains showed 3 missense mutations (D116E, K128T, V488I). In the cross‐resistant strains, 7 of 9 strains displayed 6 missense mutations, including D116E, K128T, V488I, T123I, Y132H and A516P. The missense mutation rates of each group were presented in Table 6. It is clear that the missense mutation rate of cross‐resistant strains (77.8%) was higher than that in the sensitive strains (10%), FCA‐resistant strains (37.5%), ITR‐resistant strains (44.4%), and VRC‐resistant strains (57.1%). By Fisher's exact probability analysis, there was a statistically significant difference in the missense mutation rate between the cross‐resistant strains and sensitive strains (*p* = .0452).

**Table 6:** Missense mutation rates of each group in ERG11 gene sequencing

**Group**	**Number of strains**	**Number of strains with missense mutation**	**Missense mutation rates**
Sensitive	10	1	10%
Cross‐resistant	9	7	77.8%
FCA‐resistant	8	3	37.5%
ITR‐resistant	9	4	44.4%
VRC‐resistant	7	4	57.1%

Abbreviations: FCA, fluconazole; ITR, itraconazole; VRC, voriconazole.

### 3.5 Correlation between the expression of SAP2 and as well as ERG11 and drug resistance

**Figure 3:**
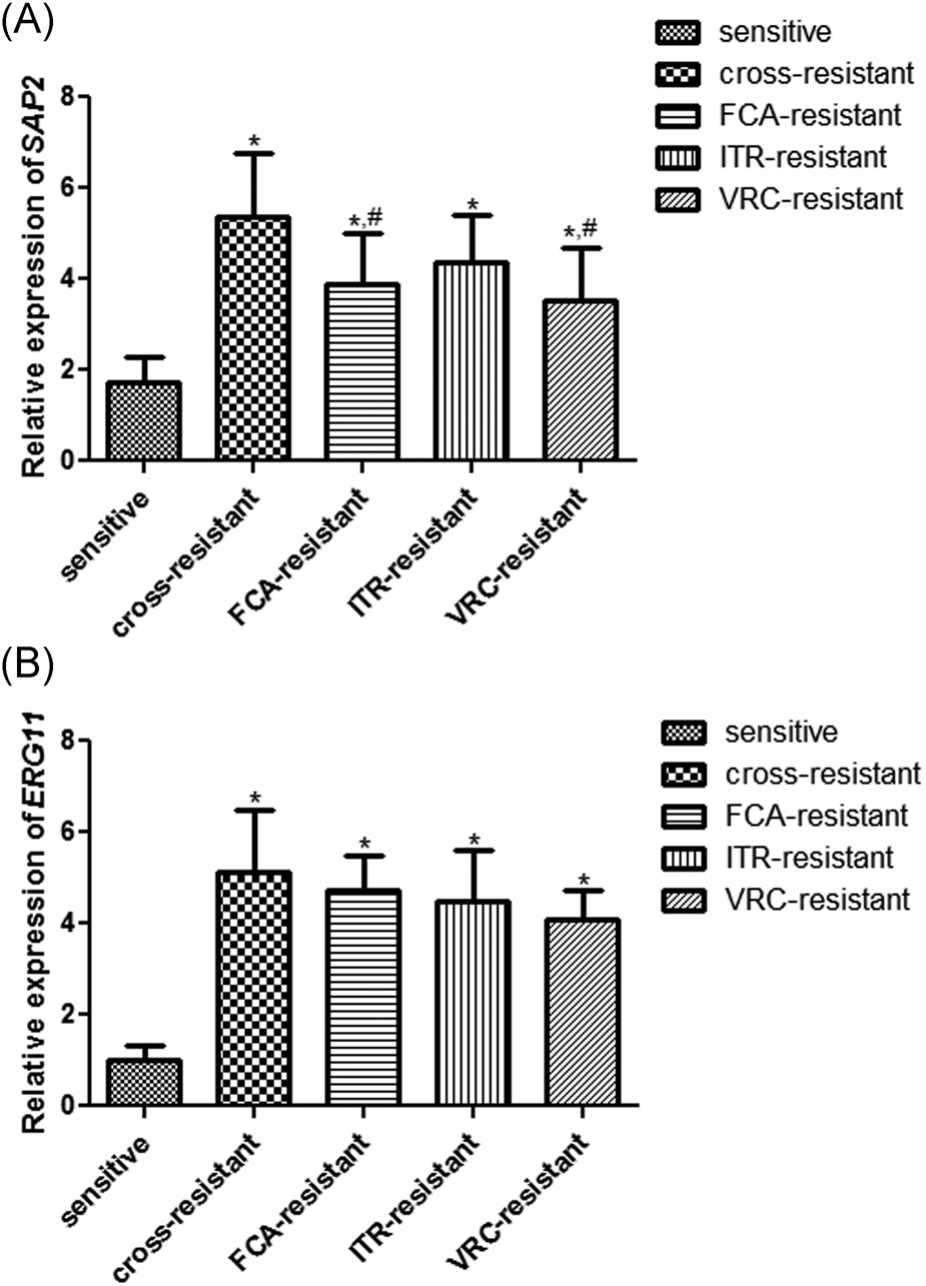
The mRNA expression of SAP2 (A) and ERG11 (B) in the sensitive, cross‐resistant, FCA‐resistant, ITR‐resistant and VRC‐resistant strains. *p < .05, compared with the sensitive strains; ^#^ *p* < .05, compared with the cross‐resistant strains. FCA, fluconazole; ITR, itraconazole; mRNA, messenger RNA; VRC, voriconazole

The expression of *SAP2* in the sensitive, cross‐resistant, FCA‐resistant, ITR‐resistant, and VRC‐resistant strains was 1.715 ± 0.576, 5.380 ± 1.39, 3.879 ± 1.125, 4.385 ± 1.02, and 3.534 ± 1.162, respectively (Figure 3A). Compared with the sensitive strains, SAP2 expression was significantly upregulated in the cross‐resistant strains and single drug resistant strains (*p* < .05). However, *SAP2* expression in the FCA‐resistant strains and VRC‐resistant strains was significantly lower than that in the cross‐resistant strains (*p* < .05). For *ERG11*, its expression was markedly higher in the cross‐resistant strains and single drug resistant strains compared to the sensitive strains (*p* < .05, Figure 3B). Additionally, there was no significant difference in the *ERG11* expression among cross‐resistant, FCA‐resistant, ITR‐resistant and VRC‐resistant strains (*p* > .05).

The results of Pearson linear correlation coefficient analysis showed that there was a positive liner correlation between *SAP2* and *ERG11* mRNA expression (*r* = .6655, *p* < .001).

## 4 DISCUSSION

*C. albicans*, the main human fungal pathogen, can cause fungal infection and seriously affect people's health and life. In this study, we found that all the 50 clinical strains formed transparent rings, and by analyzing *p*z values, the Saps activity of cross‐resistant strains was the highest, followed by the VRC‐resistant strains, FCA‐resistant strains, ITR‐resistant strains, and sensitive strains. The results of in vivo mice model showed that the pathogenicity of *C. albicans* in descending order was as follows: cross‐resistant strains, VRC‐resistant strains, ITR‐resistant strains, FCA‐resistant strains, and sensitive strains. When strains were treated with RIT, the Saps activity was inhibited with the increase of RIT concentrations. Drug sensitivity results showed that there was no synergistic effect between RIT and FCA on *C. albicans* with different drug resistance. *SAP2* sequencing showed that no gene mutation sites were found in all sequencing strains. For *ERG11* sequencing, 35 strains had base mutations, including 17 kinds of synonymous mutations and 6 kinds of missense mutations (T495A, C515T, A530C, T541C, G1609A, G1693C). Additionally, there was a positive liner correlation between *SAP2* and *ERG11* mRNA expression (*r* = .6655, *p* < .001).

In the previous study, the proteases of 37 *C. albicans*, 7 *C. glabrata*, 5 *C. parapsilosis*, and 12 *C. tropicalis* were determined, and it was found that 74.56% of the strains detected Saps, 44.73% of the strains detected PL,^24^ which indicated that the production of Saps and PL may be the important virulence factor of *Candida*. Our research showed that 50 clinical strains of *C. albicans* cultured on BSA medium formed obvious transparent rings, which proved that each strains produced Saps. Price et al.^17^ defined the *p*z value, and proposed that the smaller *p*z value was, the higher Saps activity was; *p*z value equalled to 1 meant the strains did not produce Saps. Sanitá et al.^25^ isolated *C. albicans* from the healthy subjects (HS), diabetics (DOC) and non‐diabetics with oral candidiasis (NDOC), and measured the activity of Saps, as well as found that *C. albicans* from NDOC showed the lower Saps activity compared to the *C. albicans* from HS and DOC. Our results showed that Saps activity of cross‐resistant strains was significantly higher than that in the sensitive strains. It was speculated that the Saps activity of *C. albicans* may be associated with the source of strains and their drug resistance. However, the specific reasons should be further explored.

To evaluate the pathogenicity of *C. albicans* with different drug resistance, an intravenous infection model of systemic candidiasis was established. The mice were injected with fungal suspension by tail vein, and blood culture results proved the model was successfully constructed. In the current study, the median survival time of the cross‐resistant group, FCA‐resistant group, ITR‐resistant group, VRC‐resistant group, and sensitive group was 12, 22, 18, 13.5, and 20 days, respectively. This implied that the pathogenicity of cross‐resistant strains was the highest, followed by VRC‐resistant strains, ITR‐resistant strains, FCA‐resistant strains, and sensitive strains. Schaller et al.^26^ reported that *C. albicans* lacking *SAP1* or *SAP2* could reduce tissue damage and decrease the expression of proinflammatory cytokines, which indicated that the potential for Saps to cause tissue damage may be related to epithelial‐induced proinflammatory cytokine responses. Another study showed that some Saps, particularly Sap2, could mediate the acute inflammatory response of inflammatory corpus‐dependent vaginal epithelial cells to *C. albicans*.^8^ Combined with our results, it was further proved that Saps activity may be closely related to the pathogenicity of *C. albicans*, and it was speculated that higher Saps activity in drug‐resistant strains may enhance the adhesion of *C. albicans*, promote tissue damage and inflammatory response, resulting in high mortality of mice. However, more studies are needed to be performed to investigate the potential mechanisms.

RIT has been reported to be a kind of HIV protease inhibitors, and to directly inhibit Saps of *C. albicans*.^27^ A study of Tsang et al.^28^ showed that when *C. albicans* exposed to 100 μmol/L RIT, its adhesion decreased by 50%. In our study, different concentrations of RIT were used to treat with *C. albicans*, and the *p*z values were determined. It was obvious that the Saps activity of *C. albicans* was gradually inhibited with the increase concentrations of RIT. Therefore, we hypothesized that RIT could suppress toxicity of C. albicans by inhibiting Saps activity, and RIT combined with azole drugs (FCA) may have synergistic effects on the inhibition of *C. albicans*. To verify the hypothesis, drug sensitivity experiments were carried out. The results showed that under free and biofilm conditions, RIT and FCA had no synergistic effects on *C. albicans*; the additive rates of RIT combined with FCA were respectively 16% and 24%; others displayed no interaction between FCA and RIT. Previous study has indicated that RIT decreased the growth of *C. albicans* and the activity of Saps in a nitrogen‐limited medium, the yeast carbon base and BSA (YCB‐BSA).^11^ After comparing the components of RPMI1640 medium and YCB‐BSA medium, we speculated that the reasons may be the different growing environment of *C. albicans* and the activity of Saps. Therefore, the drug sensitivity assays of RIT combined with FCA are needed to be further performed on the YCB‐BSA medium, or the synergistic effects between FCA and RIT should be confirmed through an animal model.

Saps are the primary virulence factor of *C. albicans*, and Sap2 is an important extracellular protease in the Saps family. Whether SAP2 mutates in nature and its relationship with candida albicans resistance to azole drugs are unknown. In this study, *SAP2* gene was sequenced, and it was found that there were no gene mutation sites in all sequencing strains. The possible reasons can be that SAP2 gene sequence is relatively conservative and rarely mutates, which may have nothing to do with the drug resistance of *C. albicans*. However, some scholars believed that the high expression of *SAP2* in *C. albicans* was associated with drug resistance. Copping et al.^29^ found that the expression of *SAP2* in the FCA‐resistant strains was higher than that in the sensitive strains, which indicated that *SAP2* may be related to the drug resistance of *C. albicans*. Another study demonstrated that upregulation of *SAP2* in *C. albicans* exposed to FCA may be an adaptive mechanism.^30^ Our results showed that the mRNA expression of *SAP2* was significantly upregulated in the resistant strains compared with the sensitive strains, and the *SAP2* expression was the highest in the cross‐resistant strains. Therefore, we speculated that high expression of *SAP2* may be related to the drug resistance of *C. albicans*.

Previous study has implied that the drug resistance of *C. albicans* is a complex process involving multiple mechanisms.^18^, ^31^ *ERG11* has been proved to play an important role in drug resistance. We sequenced ERG11 gene in the all clinical strains, and found 17 kinds of synonymous mutations and 6 kinds of missense mutations. These missense mutations corresponded to six different amino acids substitutions, including D116E, T123I, K128T, Y132H, V488I, A516P. D116E represented that after T495A missense mutation, the encoded amino acid was changed from aspartic acid to glutamic acid at 116th position. Our study showed that D116E was occurred not only in the sensitive strains, but also in single drug‐resistant strains and cross‐resistant strains, which indicated that D116E may not be connected with drug resistance. The results were in accordance with previous study.^32^ K128T and V488I substitutions were observed in the single drug resistant strains and the cross‐resistant strains. Wang et al.^33^ reported that the point mutation of K128T could not affect *C. albicans* resistance to FCA, which was inconsistent with our results. Thus, the relationship between K128T mutation and resistance to azoles should be further explored. Manastir et al.^34^ demonstrated that V488I mutation in *ERG11* gene were determined in the FCA‐resistant isolates, and may be associated with the azoles resistance of *C. albicans*. In addition, a study of Wu et al.^35^ indicated that substitution T123I and Y132H in *ERG11* gene could confer resistance to FCA and VRC. Our results showed that T123I and Y132H substitutions were only found in cross‐resistant strains, which hinted that T123I and Y132H mutations in ERG11 may be lead to cross‐resistance of *C. albicans* to azoles. A516P appeared in the cross‐resistant strains, and was a new mutation point that was not found in the previous papers. Consequently, further studies are needed to be carried out on the resistance of *C. albicans* to azoles caused by A516P.

In addition, the upregulation of *ERG11* in *C. albicans* can promote ergosterol synthesis, thus increasing the drug resistance. However, an in vitro study had found this resistance is reversible, suggesting that the high expression of *ERG11* may be an adaptive mechanism in the resistant strains, and not directly related to the resistance of *C. albicans* to azoles.^36^ In this study, RT‐qPCR showed that the expression of *ERG11* was markedly higher in the cross‐resistant strains and single drug resistant strains compared to the sensitive strains. The results were consistent with the previous reports.^37^ Because of the complexity of drug resistance mechanisms, we further analyzed the relationship between *SAP2* as well as *ERG11* expression and drug resistance. Pearson linear correlation coefficient analysis indicated that there was a positive liner correlation between *SAP2* and *ERG11* mRNA expression. This implied that ERG11 and SAP2 may have a certain relationship in *C. albicans*, which jointly lead to the emergence of resistance to azole drugs.

## 5 CONCLUSION

In conclusion, the Saps activity and pathogenicity of *C. albicans* in the cross‐resistant strains were the highest, and RIT could inhibit the Saps activity. However, there was no synergistic effect between RIT and FCA on *C. albicans* with different drug resistance. Additionally, the expression of *SAP2* and *ERG11* was higher expressed in the resistant strains, and there was a positive liner correlation between *SAP2* and *ERG11* mRNA expression. All these results may help to improve our understanding of azole‐resistant mechanisms of *C. albicans* and provide a novel direction for clinical therapeutics of diseases caused by *C. albicans*.

## CONFLICT OF INTERESTS

The authors declare that there are no conflict of interests.

## AUTHOR CONTRIBUTIONS

Wenli Feng and Jing Yang designed the research. Wenli Feng, Jing Yang, Yan Ma, Zhiqin Xi, and Xiaoqin Zhao did the experiment and obtained the data. Xiaoxia Zhao and Min Zhao analyzed and explained the data. Wenli Feng and Jing Yang drafted the manuscript, and Wenli Feng revised. All authors have read and approved the final version.

## Data Availability

The datasets used and/or analyzed during the current study are available from the corresponding author on reasonable request.
